# Preventing Urgent Pediatric Readmissions: The Need for and Promise of Real-Time Monitoring

**DOI:** 10.2196/60802

**Published:** 2025-06-23

**Authors:** Isha Thapa, Brian Han, David N Rosenthal, Stephen J Roth, Andrew Y Shin, Nicholas Bambos, David Scheinker

**Affiliations:** 1 Department of Management Science and Engineering, Stanford University, 475 Via Ortega, Stanford, CA, 94305, United States; 2 Division of Pediatric Cardiology, Stanford University School of Medicine, Stanford, CA, United States; 3 Department of Electrical Engineering, Stanford University, Stanford, CA, United States; 4 Division of Pediatric Endocrinology, Stanford University School of Medicine, Stanford, CA, United States; 5 Clinical Excellence Research Center, Stanford School of Medicine, Palo Alto, CADivision of Endocrinology, Department of Pediatrics, Stanford University School of Medicine, CA, United States; 6 Division of Hospital Medicine, Department of Medicine, Stanford University School of Medicine, CA, United States

**Keywords:** medical information, remote patient monitoring, RPM, telehealth, pediatric readmissions, patient care management, chronic condition, readmission, disease management, intervention

## Abstract

Urgent pediatric hospital readmissions are common, costly, and often preventable. Existing prediction models, based solely on discharge data, fail to accurately identify pediatric patients at-risk or urgent readmission. Remote patient monitoring (RPM) leverages wearable technology to provide real-time health data, enabling care teams to detect and respond to early signs of clinical deterioration. Emerging evidence suggests RPM may be a promising strategy to improve pediatric postdischarge outcomes and reduce urgent hospital readmissions.

## Introduction

Untreated clinical deterioration in pediatric patients with chronic conditions can lead to urgent hospital readmissions, impacting the health of children, imposing a significant burden on health care systems, and delaying the care of others [1,2]. The rates of pediatric urgent readmissions, unplanned hospitalizations occurring within 30 days of discharge that require immediate medical intervention, vary from 3% to 19% across pediatric hospitals and up to 40% of them may be preventable [2]. The data available at discharge have not proved useful for predicting readmissions, especially for postdischarge causes such as medication-related failures [3]. Remote patient monitoring (RPM) may be a viable approach to detecting the early stages of clinical deterioration that ultimately lead to urgent readmission. A variety of wearable monitors enable the timely identification of deteriorating disease management and interventions to remediate it [4-6]. Used appropriately, wearable technology has the potential to help care teams reduce urgent readmissions. We argue that care models that leverage RPM have the most potential to allow care teams to reduce urgent hospital readmission for patient with chronic disease by identifying complications that may otherwise escalate into serious health concerns.

## Challenges in Predicting Readmissions

Several studies have shown that predicting urgent pediatric hospital readmissions at the time of discharge is challenging, particularly for general- and for complex-subspecialty patient hospitalizations [2]. Initial efforts in this domain have concentrated on the identification of risk factors using odds ratios from data available at the time of discharge [3]. However, few of these studies have been externally validated or demonstrated to reduce readmissions in practice.

A systematic review of predictive models for 30-day unplanned pediatric readmissions found poor-to-moderate performance across most models [2]. Of the 37 models reviewed, all models except one had an area under the curve (AUC), a flawed but broadly recognized benchmark, below 0.8. The review assessed the quality of these studies to be moderate to low across all domains. Most studies lacked adequate representation of the broader pediatric population, often examining patients from only a single hospital. Many studies lacked sufficient consideration of potential confounders in their analyses.

Tools for predicting unplanned readmissions, such as the High Acuity Readmission Risk Pediatric Screen Tool, LACE (Length of stay, Acuity of admission, Comorbidity, Emergency department use), LACE-SDH (which adds Social Determinants of Health), LACE+, Epic’s readmission risk model, and SQLAPE demonstrate only moderate performance, with AUCs ranging from 0.61 to 0.80 [7]. These modest performance metrics likely overstate the usefulness of such models for targeting limited resources to the relatively few patients with high risk of readmission [8]. Existing models likely fail to predict pediatric readmissions effectively because they rely on static data captured at the time of discharge, which does not reflect the evolving nature of recovery. Moreover, many are based on simple scoring systems or regression models that may inadequately account for the complexity of pediatric health trajectories.

It is not surprising that data available at the time of discharge are not sufficient to predict pediatric readmissions. Posthospitalization care is complex and lacks robust quality standards. Up to one-third of pediatric discharges may be associated with discharge-related care failures. Medication adherence serves as a prime example. A study of 157 caregivers revealed that 70% encountered medication-related failures [9]. These findings highlight the pressing need for improved postdischarge monitoring and/or interventions.

## RPM

RPM offers promise for preventing readmissions. It is a technology-enabled approach to precision population health that facilitates the continuous capture and transmission of patient health metrics to medical practitioners when patients are not in the clinic. Portable devices have been validated for monitoring activity levels, sleep quality, heart rate, oxygen saturation, blood pressure, and glucose levels. Data from portable devices are often used to help manage chronic disease and detect deteriorating health in pediatric and adult populations [4-6,10,11]. Remote data acquisition and algorithm-supported analyses enable clinicians to detect health deterioration and adverse symptoms or health patterns when patients are not in the clinic. This enables timely investigation by the care team and, when appropriate, interventions to prevent the escalation of minor health concerns into serious complications.

Evidence for the effectiveness of RPM is already emerging in specific pediatric populations. An RPM intervention using continuous glucose monitors (CGMs) in pediatric patients with type 1 diabetes led to significant improvements in glucose control [6]. A small prospective study on pediatric patients receiving automated peritoneal dialysis resulted in a 45% reduction in hospitalization rates, significant increase in ultrafiltration, and significant reduction in systolic blood pressure [12]. RPM for children with single ventricle heart disease is now supported by the American Heart Association, after RPM contributed to mortality reductions of over 40% over an 8-year period [13]. RPM can also lead to more efficient workflows for health care providers, potentially reducing the time spent on routine checkups. For example, the conjunction of CGM with an automated tool was associated with improved outcomes and reduced provider screen time [6,14].

Health care systems adopting RPM will need to consider how to integrate workflows into existing clinical systems [15]. Effective implementation may include developing standardized protocols for alert management, integrating multiple monitoring devices into unified systems, and clearly defining care team responsibilities for responding to patient data. Implementing interoperability features that generate reports for inclusion into patients’ medical records and import monitoring statistics into electronic medical record flowsheets can streamline clinical workflows [15]. A successful example comes from a CGM-based intervention that used a dashboard to prioritize patients for review, which was later adapted into a nationally available platform for broader adoption [15]. When an RPM system demonstrates efficacy within a single clinic, the standardized approach can facilitate expansion across multiple health care settings, creating opportunities for wider implementation and continuous improvement in patient care.

While RPM offers significant potential, barriers to access may affect its equitable implementation. Digital disparities, including limited internet access and device availability, could restrict RPM use [6,11]. Approximately 20% of US households lack access to a smartphone. In addition, variation in caregiver technological proficiency may affect adherence to monitoring protocols, and limited digital literacy can influence program engagement [11]. Addressing these challenges requires consideration of technology accessibility, device subsidization, user-friendly interfaces, and culturally appropriate education [6,11]. RPM programs “should be designed to accommodate populations with low health literacy and numeracy, with language preferences in mind” [11].

Proof-of-concept studies are necessary to determine how the implementation of RPM can reduce pediatric readmissions in the real clinical setting. Initial studies should focus on the assessment of technical feasibility within existing health care systems along with thoughtful mapping of how RPM would integrate into the clinical workflow of the care of the chronic pediatric patient. As these studies scale, outcome measures should capture not only clinical outcomes, but also include patient-reported outcomes and cost-effectiveness [16]. Pilot studies can then inform the design of larger randomized controlled trials comparing standard discharge protocols to those enhanced with RPM and allow scaling of its implementation [17].

## Conclusions

Urgent pediatric hospital readmissions are common and preventable. Existing readmission prediction models are not sufficient to identify the patients at highest risk, especially those associated with care failures. RPM is a promising tool that offers real-time data transmission, streamlined operational procedures, and scalability. As RPM technology becomes more accessible, its potential to transform pediatric postdischarge care and reduce urgent readmissions warrants further investigation. Advancing this work may enable more proactive and effective strategies to improve health outcomes and reduce urgent readmissions.

## Abbreviations

AUC: area under the curve
CGM: continuous glucose monitor
LACE: Length of stay, Acuity of admission, Comorbidity, Emergency department use
LACE-SDH: Length of stay, Acuity of admission, Comorbidity, Emergency department use–Social Determinants of Health
RPM: remote patient monitoring
